# Adverse Childhood Experiences and Early Maladaptive Schemas as Predictors of Cyber Dating Abuse: An Actor-Partner Interdependence Mediation Model Approach

**DOI:** 10.3389/fpsyg.2021.623646

**Published:** 2021-03-18

**Authors:** Laura Celsi, F. Giorgia Paleari, Frank D. Fincham

**Affiliations:** ^1^Department of Human and Social Sciences, University of Bergamo, Bergamo, Italy; ^2^Family Institute, Florida State University, Tallahassee, FL, United States

**Keywords:** cyber dating abuse, ACEs, early maladaptive schemas, actor-partner interdependence mediation model, romantic couples, young adults

## Abstract

The increasing role that new technologies play in intimate relationships has led to the emergence of a new form of couple violence, cyber dating abuse, especially among adolescents and young adults. Although this phenomenon has received increased attention, no research has investigated predictors of cyber dating abuse taking into account the interdependence of the two partners. The study examines adverse childhood experiences (abuse, neglect, and witnessed intimate partner violence) and early maladaptive schemas (emotional deprivation and abandonment) as possible predictors of young adults’ perpetrated and suffered cyber dating abuse. Adopting a dyadic approach, mediational models in which adverse childhood experiences were assumed to be related to individual and partner’s cyber dating abuse through individual early maladaptive schemas were tested. 134 couples completed online self-reports of the variables of interest, including a bidimensional measure of cyber dating abuse assessing pressure-aggression and control-monitoring. Actor-partner interdependence mediation model analyses were conducted. Results indicated that the emotional deprivation schema mediated the association between adverse childhood experiences and cyber dating abuse, whereas the abandonment schema did not. Specifically, more frequent experiences of emotional abuse and physical neglect during childhood were indirectly related to increased likelihood of perpetrating cyber dating pressure-aggression as well as of perpetrating and suffering cyber dating control-monitoring in both males and females. These associations were mediated by a stronger internalization of the emotional deprivation schema and were supported by both self-reported and partner-reported data. Also, a strong and direct association was found between childhood exposure to intimate partner violence by the opposite-sex parent and cyber dating pressure-aggression by females or control-monitoring by both males and females. These findings help to clarify the potential negative effects of specific adverse childhood experiences and early maladaptive schemas on the tendency to perpetrate and suffer cyber abuse in romantic relationships. The implications for prevention and treatment programs are noted and avenues for future research are described.

## Introduction

The couple is a third physical and psychic entity that emerges at the meeting place between two worlds, embodied by the individual partners who constitute it. Due to the increasing role that new technologies play in our society, especially among adolescents and young adults, the meeting between partners, the construction of the couple, and even the implementation of dysfunctional dynamics damaging to individual health and relationship quality, take place online as well as offline.

Thus, more and more frequently people tend to use social networks or messaging apps to deepen their knowledge of potential partners. This is because the distance given by social networks is perceived to facilitate the disclosure of one’s feelings, reduce problems caused by one’s shyness, and limit the sadness and the sense of defeat deriving from a possible refusal. Similarly, once the couple is created, new technologies are often the medium used not only to maintain dating relationships, but also to express one’s anger toward the partner or verify the trust given to him/her through digital control (Burke et al., 2011). This often entails negative and dysfunctional behaviors, such as falsifying one’s identity, engaging in aggressive acts, and violating privacy, which can be more easily enacted online than offline due to the higher levels of detachment and self-centeredness and lower levels of empathy and accountability which characterize online interactions (Drauker and Martsolf, 2010; Runions and Bak, 2015).

For this reason, understanding couple violence among adolescents and young adults necessarily involves investigating Cyber Dating Abuse (CDA). CDA is an emerging form of abuse which consists of using mobile phones and digital social networks to control the partner, limit his/her freedom, mock, denigrate, threaten, and/or force him/her to perform or suffer unwanted sexual acts (Zweig et al., 2013, 2014; Borrajo et al., 2015a; Reed et al., 2016) for a review see Caridade et al. (2019). This phenomenon is becoming a public health issue, as existing studies (e.g., Borrajo et al., 2015b; Reed et al., 2016, 2017) have found victimization and perpetration of CDA in at least 50% of participants. Italian data do not differ, as the only study in Italy documented the presence of psychological violence online in 60% of adolescents and young adults (Morelli et al., 2018).

Some data show that CDA is related to but distinct from Intimate Partner Violence (IPV) due to the characteristics of the medium through which CDA occurs (e.g., Zweig et al., 2013; Dick et al., 2014; Borrajo et al., 2015a; Marganski and Melander, 2015; Sargent et al., 2016; Temple et al., 2016; Deans and Bhogal, 2019). Regarding abuse perpetration, for example, digital technologies make it easier to commit violent acts and reduce social emotional cues, which may elicit less empathy and greater violence. Concerning victimization, the fact that violence can be carried out continuously increases the victim’s perception of vulnerability. Furthermore, the possibility that CDA is perpetrated in a public domain, characterized by the persistence of written or posted content, increases exponentially the negative effects of the damage suffered and raises the probability of re-victimization experiences (Bennet et al., 2011; Lucero et al., 2014; Borrajo et al., 2015a,b,c; Peskin et al., 2017). For this reason, more studies are needed both to better understand CDA and to clarify whether it is a new form of violence or simply an evolution of IPV.

Like any phenomenon in a dyadic relationship, CDA is linked to individual traits and experiences as well as to relational dynamics, manifested as a series of emergent characteristics, which arise from the encounter between the subjectivities of the two partners. Thus, in relationships, individual behaviors are not only determined by intra-individual characteristics and events but undergo a series of modulations that depend on the couple (Framo, 1992). In particular, although personality traits or personal experiences can predispose a person to perform certain behaviors, the partner can facilitate or inhibit these behaviors, depending on his or her own personality traits and experiences. Therefore, with specific reference to the study of CDA, knowing the predisposition of each partner toward the perpetration of this form of violence could be insufficient, because it does not take into account the variations that the behavior of one partner could undergo depending on the behavior of the other partner. Thus, for example, a person with a low predisposition to control could be induced to implement online controlling behaviors if involved in a relationship with a particularly secretive partner and therefore be capable of instilling feelings of jealousy and fear of betrayal. Similarly, a person not particularly predisposed to submission could be consciously the victim of online control by a partner who, moved by an excessive fear of betrayal and abandonment, tends to evaluate as negative and dangerous his or her partner’s attempts to establish an appropriate level of autonomy in the relationship. To the best of our knowledge, CDA has not been investigated from such a dyadic perspective. However, a few studies have examined IPV as a function of both partners’ characteristics like adult attachment styles, borderline personality traits, and the perceived fulfillment of basic psychological needs (Maneta et al., 2013; Sommer et al., 2016; Petit et al., 2017). Therefore, one goal of our study is to consider the role played by individual variables in predicting involvement in violent relationships while taking into account the interdependence between the partners.

Research on CDA etiology is in its infancy, but evidence on IPV, to which CDA is related, suggests that adverse childhood experiences (ACEs) and early maladaptive schemas may be distal predictors of CDA.

ACEs, defined as negative, stressful, and traumatic experiences during childhood and adolescence (Felitti et al., 1998), are undoubtedly some of the most studied risk factors for involvement in violent relationships. This construct is multidimensional and includes multiple traumatic experiences, which impair the psycho-physical development of the individual, including physical abuse, sexual abuse, emotional abuse, physical neglect, emotional neglect, witnessed violence, cohabitation with a family member suffering from psychiatric pathologies or addiction to alcohol or drugs, and the imprisonment of a family member (Felitti et al., 1998; Anda et al., 2002; Bernstein et al., 2003; Chapman et al., 2004; World Health Organization (WHO), 2018). Among these negative experiences, the most studied as predictors of IPV are the various forms of abuse and neglect and, in recent years, witnessed violence. The overwhelming majority of research has found that all forms of abuse, neglect, and witnessed violence increase the likelihood of victimization and perpetration of violence within couples (Whitfield et al., 2003; Garrido and Taussig, 2013; Karakurt et al., 2013; Iverson et al., 2014; Eriksson and Mazerolle, 2015; McMahon et al., 2015; Machisa et al., 2016; Madruga et al., 2017; Voith et al., 2017; Kimber et al., 2018; Li et al., 2020; Yan and Karatzias, 2020).

Early maladaptive schemas constitute the fundamental construct of Schema Therapy, an epistemological and psychotherapeutic model developed by Young and colleagues in the 1990s with the goal of making cognitive-behavioral therapy (CBT) more suitable for treating people with pathological traits or personality disorders. According to Young et al. (2007), schemas are dysfunctional emotional and cognitive structures that people use to understand and give meaning to oneself, to others, and to the events that occur. These patterns arise during childhood and adolescence based on somatic sensations, emotions, memories, and thoughts connected to experiences. The circumstances that favor the emergence and consolidation of maladaptive schemas are the frustration of at least one of the following five fundamental human needs: (1) need for stable ties with other people (need for protection, stability, care, and acceptance); (2) need for autonomy, sense of competence, and identity; (3) need to be able to freely express needs and emotions; (4) need for spontaneity and play; and (5) need for realistic limits and self-control. Young et al. (2007) identified 18 schemas, classifiable into five categories or “domains,” depending on the fundamental frustrated need to which they are linked. These domains are the following: (1) Disconnection and rejection, (2) Impaired Autonomy and/or Performance, (3) Other-Directedness, (4) Over vigilance/Inhibition, and (5) Impaired Limits. More recently, Bach et al. (2018) have found support for a model with four domains: (1) Disconnection and rejection, (2) Impaired autonomy and performance, (3) Excessive responsibility and standards, and (4) impaired limits. IPV research has shown that the domain most commonly connected to the experiences of victimization and perpetration of couple violence is Disconnection and rejection (Gay et al., 2013; Falahatdoost et al., 2014; Atmaca and Gencoz, 2016; Taşkale and Soygüt, 2017; Calvete et al., 2018; Borges and Dell’Aglio, 2020). This domain describes people unable to build lasting, safe, and fulfilling relationships because they are always convinced that others will not be able to satisfy their needs for stability, security, care, love, and acceptance. In response to patterns belonging to this domain, people may adopt maladaptive styles of coping based on overcompensation, which cause them to establish a morbid bond with their partner and experience any estrangement as dangerous due to excessive jealously and fear of betrayal and abandonment. The theory of early maladaptive schemas (Young and Flanagan, 1998) holds that they are found in people who grew up in families which were unstable (abandonment/instability), violent (distrust/abuse), inadequately affectionate (emotional deprivation), overly demanding (inadequacy/shame), or socially isolated (social exclusion) and who often suffered real trauma. This etiological explanation links the internalization of the schemas belonging to the Disconnection and rejection domain to childhood and adolescent experiences marked by adverse experiences like ACEs and gives rise to the hypothesis that early maladaptive schemas may mediate the relationship between ACEs and IPV. In line with this reasoning, Gay et al. (2013) showed that the schemas in the Disconnection and rejection domain mediate the relationship between emotional abuse and victimization or perpetration of IPV.

To our knowledge, little research has analyzed whether predictors and mediators of IPV play a similar role in CDA. Only two studies investigate the link between ACEs and CDA (Smith-Darden et al., 2016; Ramos et al., 2017). Both yielded results consistent with those relating to IPV; they showed that physical, sexual, and psychological abuse, family aggression, and family problems (e.g., family member incarceration, family member drug or alcohol use, family member mental illness) were connected with a greater likelihood of perpetrating CDA (Smith-Darden et al., 2016; Ramos et al., 2017). No study has attempted to document a connection between maladaptive schemas belonging to Disconnection and Rejection and CDA or a possible mediating role of Disconnection and rejection maladaptive schemas in the relationship between ACEs and CDA. Any attempt to address these issues must take into account the interdependence of the two partners, and their dual role as possible perpetrators and victims. Such an approach is critical for at least three reasons. First, it offers the important advantage of taking into account the predictive effects that schemas and, indirectly, ACEs have on CDA both within and across partners. As we have previously argued, CDA, like most of the dynamics occurring in a couple relationship, is likely to be perpetrated and suffered depending not only on one’s own characteristics and experiences, but also on those of the romantic partner. In line with this argument, Maneta et al. (2013) showed that offline perpetration of psychological aggression was predicted by both partners’ attachment avoidance and that offline perpetration of sexual coercion was influenced by both partners’ attachment anxiety. Therefore, a dyadic approach promises to offer a more comprehensive explanation of the phenomenon investigated. Second, many CDA studies have assumed gender asymmetries to justify the investigation of victimization only among females and of perpetration only among males. Not infrequently, however, levels of perpetration by females were similar and, in the context of control, sometimes even higher than those of males (Burke et al., 2011; Kellerman et al., 2013; Zweig et al., 2013; Borrajo et al., 2015a; Reed et al., 2017. Third, comparing the experiences of the two partners with respect to perpetration and victimization can show patterns of opinions and experiences (e.g., reciprocity, convergence, complementarity, contrast) which are fundamental to understanding well-being at the level of the couple. In line with this, Reed et al. (2017) found that females show a more tolerant attitude toward monitoring and males do the same with sexting behaviors, but within an overall negative perception of CDA. Furthermore, females experience any form of CDA suffered (monitoring, direct aggression, sexual cyber abuse) in a more negative way than males.

**FIGURE 1:**
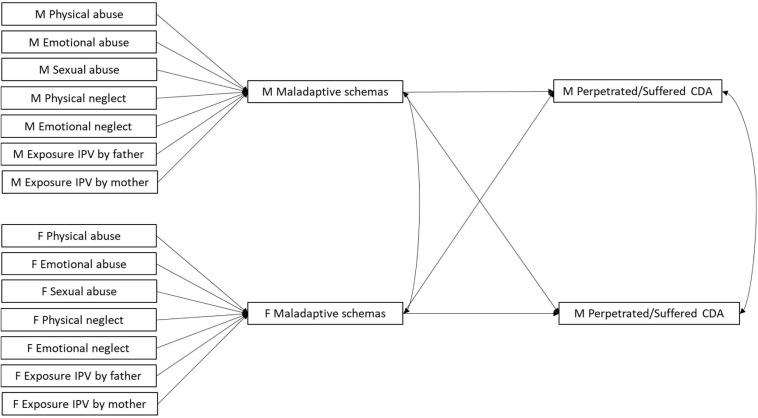
The hypothesized Actor-Partner Interdependence Mediation model (APIMeM). M = males; F = females.

Consequently, our study investigated whether ACEs predict both perpetrated and suffered CDA through the mediation of Disconnection and Rejection maladaptive schemas. Because of partner interdependence, maladaptive schemas were expected to predict CDA both within and across partners. In order to take into account the interdependence between partners, the hypothesized mediational models were tested by using the couple as the unit of analysis and by simultaneously estimating individual and partner effects using the Actor-Partner Interdependence Model (APIM; Kenny et al., 2006). Individual or actor effects refer to the effects a respondent’s predictors have on his/her outcomes. Partner effects refer to the effects of one partner’s predictors on the other partner’s outcomes and represent the interdependence that exists between the dyad members (Kenny et al., 2006). Specifically, the Actor-Partner Interdependence Mediation Model (APIMeM) tested (see Figure 1) posits both actor and partner effects from maladaptive schemas to CDA dimensions, consistent with the IPV literature previously reviewed showing that early maladaptive schemas belonging to the Disconnection and rejection domain might predict perpetrated and suffered CDA both within and across partners (Gay et al., 2013; Falahatdoost et al., 2014; Ramos et al., 2017; Taşkale and Soygüt, 2017). However, the model posits only actor effects from ACEs to maladaptive schemas because, according to the theory of early maladaptive schemas (Young and Flanagan, 1998), the construction of maladaptive schemas is directly connected to early personal experiences which are likely to precede meeting the romantic partner. Thus, the APIMeM proposed by Ledermann et al. (2011) was adapted so that partner effects were hypothesized only from the mediating to the outcome variables, and not from the independent to the mediating variables.

Informed by the IPV literature, the following hypotheses were examined. First, the more subjects had experienced forms of abuse and neglect in their family of origin and had been exposed to intimate partner violence enacted by their parents, the more: (a) they were likely to have developed early disconnection and rejection schemas, according to which others are perceived as unable to satisfy their needs for stability, security, care, love, and acceptance; and (b) they themselves as well as their romantic partner were likely to perpetrate and suffer CDA (see Eriksson and Mazerolle, 2015; Smith-Darden et al., 2016; Ramos et al., 2017; Calvete et al., 2018; Borges and Dell’Aglio, 2020). Second, early maladaptive schemas mediate the link between ACEs and CDA, so that, independently of ACEs, the more subjects developed early disconnection and rejection schemas the more likely they and their partner were to perpetrate and suffer CDA (see Gay et al., 2013; Corral and Calvete, 2014; Falahatdoost et al., 2014; Taşkale and Soygüt, 2017).

## Materials and Methods

### Participants

One hundred seventy-eight non-cohabiting couples took part in the study. However, 29 couples were excluded from the analyses because one or both partners had omitted some answers. Furthermore, since only 15 of the 149 remaining couples were made up of homosexuals and bisexuals, we considered this group too small to be compared with that of heterosexual couples. Thus, our final sample comprised 134 non-cohabiting heterosexual couples.

Participants were almost exclusively white (99.3% of males, 100% of females), Italian (95.5% of males, 98.5% of females), and mostly resided in northern Italy (51.5% of males, 49.3% of females). The average age was 23.49 (SD = 2.88; range: 18–30) for males and 21.89 (SD = 2.57; range: 18–29) for females. The most frequent educational qualifications were high school diploma or equivalent (56.7% of males, 59% of females) and bachelor degree (23.9% of males, 22.4% of females). In terms of working condition, the majority of females were students (63.4%), while most males were students (41%) or full-time workers (25.4%). The average daily number of hours spent on the internet, social networks, and messaging applications was 4.14 (SD = 2.33; range: 0.75–13) for males and 3.51 (SD = 0.33–13.67) for females. Finally, the couple relationship in which the participants were involved averaged about 3 years (M = 33.2 months; SD = 26.12; range: 3–118).

### Procedure

Participants were contacted through the publication of a post on the walls of university groups registered on Facebook. The message presented the study as one on the impact of new technologies on late adolescent and young adult couple relationships and informed participants about the anonymous nature of the survey. It also specified the inclusion criteria (being aged between 18 and 30, having a romantic relationship lasting at least three months, not living with the partner), the average response time (approximately 30 min), and how to fill in the survey (each partner would have to answer the questions individually). The data collection was limited to non-cohabiting partners because dating relationships are more common among Italian adolescents and young adults. We also chose to focus on romantic relationships lasting at least 3 months because understanding the effect of mutual influence between partners’ maladaptive schemas requires couples who have had the time to build sufficiently consolidated and recursive relational dynamics. Finally, the post contained the link to an online survey and thanked the participants for their collaboration. Before completing the survey, all respondents reviewed, signed, and submitted an informed consent form. All participants were treated according to the ethical guidelines established by the Italian Psychological Association (AIP, 2015). These guidelines include obtaining informed consent from participants, maintaining ethical treatment and respect for their rights, and ensuring the privacy of participants and their data.

### Measures

#### ACEs

The adverse childhood experiences were measured through five subscales from the Italian version of the Childhood Trauma Questionnaire – Short Form (CTQ-SF; Bernstein et al., 2003; Petrone et al., 2012; Sacchi et al., 2018), which is one of the most used retrospective instruments for detecting adverse experiences in the family of origin during childhood or adolescence, as well as through six *ad hoc* items assessing violence witnessed in the family during the same period. The CTQ-SF subscales measure: physical abuse (five items; e.g., “People in my family beat me so hard they left bruises or marks on me”), emotional abuse (five items; e.g., “People in my family used to offend and insult me”), sexual abuse (five items; e.g., “Someone tried to get me to do sexual things or watch sexual things”), physical neglect (five items; e.g., “I didn’t have enough to eat”), and emotional neglect (five items; e.g., “People in my family felt very close” – reverse item).

The six *ad hoc* items assessed physical and psychological IPV perpetrated by parents and witnessed by respondents (e.g., “I saw/heard my mother being insulted, denigrated, humiliated, or verbally assaulted by my father”). In line with Eriksson and Mazerolle (2015), who verified that the effects of witnessed violence can change according to the gender of the child and to that of the abusive parent, three items measured violence perpetrated by the father against the mother and three items assessed violence perpetrated by the mother against the father. From here on, we will refer to these two forms of violence respectively as “exposure to IPV acted by the father “and “exposure to IPV acted by the mother.”

Participants were asked to respond to all items by reporting on a 5-point Likert scale how many times the behavior described had occurred during childhood and adolescence (1 = never, 2 = seldom, 3 = sometimes, 4 = often, 5 = very often).

All scales showed good reliability (physical abuse: α = 0.78 for males and 0.70 for females; emotional abuse: α = 0.88 and 0.92; sexual abuse: α = 0.88 and 0.88; physical neglect: α = 0.79 and 0.71; emotional neglect: α = 0.89 and 0.79; exposure to IPV acted by the father: α = 0.82 and 0.87; exposure to IPV acted by the mother: α = 0.79 and 0.80).

We excluded sexual abuse from our analyses because it was rarely experienced in our sample (4% of women and 7% of men).

### Early Maladaptive Schemas Belonging to the Disconnection and Rejection Domain

Early maladaptive schemas belonging to the Disconnection and rejection domain were measured through the Italian version of the Young Schema Questionnaire Short Form 3 (YSQ-S3; Young, 2005; Baldetti et al., 2015), which asks subjects to use a 6-point Likert scale, ranging from “Completely false for me,” to “It describes me perfectly,” to express their degree of agreement with 13 statements.

The early maladaptive schemas that the theory and the aforementioned questionnaire assessed are: Abandonment, Mistrust/abuse, Emotional deprivation, Defectiveness/shame, and Social Isolation/alienation. However, the analyses of the internal structure of the scale, previously carried out on 263 Italian young adults (Celsi, 2019, unpublished), yielded only two of the five hypothesized factors for this domain: emotional deprivation, including four items (e.g., “I didn’t have anyone to look after me, open up to me, or cared deeply for whatever happened to me”; α = 0.84) and abandonment, including three items (e.g., “I find myself clinging to the people I’m close to because I’m afraid they’ll leave me”; α = 0.84). Consequently, only items belonging to the abandonment and emotional deprivation dimensions were retained in the present study. The two dimensions, which were moderately correlated (r = 0.33 for males and 0.35 for females), showed good internal consistency in the present study (emotional deprivation: α = 0.84 for females and 0.81 for males; abandonment: α = 0.80 for both females and males).

#### Perpetrated and Suffered Cyber Dating Abuse

Perpetrated and suffered CDA within the current romantic relationship was measured using 40 items (20 for perpetration and 20 for victimization, e.g., “I pressured my partner to have sex or engage in sexual activity with me via webcam” and “My partner pressured me to have sex or engage in sexual activity with him/her via webcam”). 22 were created for this study, 12 were derived from the Reed et al. (2017) scale, and six were taken from the Cyber Dating Abuse Questionnaire (Borrajo et al., 2015a). Both the Reed and Borrajo instruments, two of the most used CDA measures, have shown poor psychometric properties when applied to Italian samples (Celsi, 2019, unpublished), therefore necessitating use of a new CDA questionnaire. The new set of items collected information about various types of CDA: aggression, threats, control, privacy intrusion, identity theft, and pressure for sexual behaviors or for sharing sexual images. Participants were asked to report on a 7-point Likert scale how many times the behavior described by each item had occurred in the relationship with the romantic partner (0 = never, 1 = one time, 2 = two times, 3 = between three and five times, 4 = between six and ten times, 5 = between eleven and twenty times, 6 = more than twenty times).

Both the new set of items and the rating scale were refined on the basis of a pilot study that tested the discriminative power of each item on a sample of 216 young adults. Exploratory and confirmatory factor analyses, which were conducted on an additional 263 young adults using a polychoric matrix due to the non-normal item distribution, revealed two correlated factors: one including 11 items assessing cyber monitoring and control (e.g., “I/my partner looked at private information to check up on my partner/me without permission,” “I/my partner checked my/my partner’s location and online activities”) and one including nine items measuring psychological or sexual pressure and aggression (e.g., “I/my partner sent a threatening message to my partner/me,” “I/my partner pressured my partner/me to have sex or engage in sexual activity with him/her/me via webcam.” The two-factor solution was confirmed in the present study (CDA perpetrated by males: S-Bχ^2^(169) = 202.762, *p* = 0.039, R-CFI = 0.992, R-RMSEA = 0.039; CDA perpetrated by females: S-Bχ^2^(169) = 270.662, *p* = 0.000, R-CFI = 0.989, R-RMSEA = 0.067; CDA suffered by males: S-Bχ^2^(169) = 218.337, *p* = 0.006, R-CFI = 0.995, R-RMSEA = 0.047; CDA suffered by females: S-Bχ^2^(169) = 44.391, *p* = 1.000, R-CFI = 1.000, R-RMSEA = 0.000). Factor loading were all greater than 0.50 and 14 out of 20 items were invariant across males and females^1^ (perpetrated CDA: S-Bχ^2^(748) = 853.027, *p* = 0.005, R-CFI = 0.994, RMSEA = 0.033; suffered CDA: S-Bχ^2^(750) = 446.320, *p* = 1.000, R-CFI = 1.000, R-RMSEA = 0.000). The internal consistency was very good for all dimensions (perpetrated control-monitoring: α = 0.91 and 0.94 for males and females, respectively; perpetrated pressure-aggression: α = 0.95 and 0.95; suffered control-monitoring: α = 0.95 and 0.92; suffered pressure-aggression: α = 0.95 and 0.95).

### Data Analysis

Preliminary analyses were conducted, including multiple regression analyses in which all investigated ACEs were regressed on other study variables. Only ACEs that were uniquely and significantly related with the self-reported mediators or self-reported/other-reported outcomes for either males or females were included in the mediational models.

Eight APIMeMs were then tested: in four of them emotional deprivation was assumed to mediate the relationships between ACEs and pressure-aggression or control-monitoring, either perpetrated or suffered by males and females, whereas in the other four models abandonment was posited to mediate the same links. All exogenous variables in a model (i.e., ACEs) were allowed to correlate. To estimate these models, we used structural equation modeling with measured variables (EQS6.4; Bentler, 2008). Inspection of Mardia’s (1970) coefficients suggested significant deviations from multivariate normality; to reduce the impact of non-normality we relied on Satorra and Bentler (2001) scaled estimates in rescaling the standard errors and the chi-square statistics into the Satorra–Bentler scaled chi-square (S–B χ^2^) statistic. Fit indexes, like the comparative fit index (CFI) and the root-mean-square error of approximation (RMSEA), were also adjusted for non-normality by incorporating the S–B χ^2^ into their calculations. We refer to them as robust estimates (i.e., R-CFI, R-RMSEA).

Before estimating APIMs, the study variables were standardized with means and standard deviations computed across males and females so as to have coefficients comparable across dyad members (Kenny et al., 2006). To evaluate whether individual and partner effects differed across dyad members, we constrained the four individual and the two partner parameters to be equal and then assessed the degree to which each constrain worsened the fit of the model via a χ^2^ difference test (S-B Δ*χ*^2^). In case of a non-significant Δ*χ*^2^, the path was held equal across dyad members for model parsimony.

A bootstrapping procedure was used to estimate and test the indirect effects due to their non-normal distributions (Preacher and Hayes, 2008). The multivariate Lagrange Multiplier (LM) test (Bentler, 2008) was used to determine whether our full mediation models provided a better fit to the data than alternative partial mediation models, in which direct paths from ACEs to CDA dimensions were added.

Finally, to provide further support for our hypothesized APIMeMs, alternative models in which outcomes and mediators were reversed were also tested. In fact, considering the cross-sectional nature of our data and the fact that young adulthood is not so far from the childhood and adolescence period during which early maladaptive schemas are supposed to arise, it cannot be ruled out *a priori* that CDA may mediate the links between ACEs and maladaptive schemas. The hypothesized models were compared to the alternatives using the robust Akaike Information Criterion (R-AIC; Akaike, 1973; Burnham and Anderson, 2002). When comparing non-nested models estimated from the same data, the model with the smaller AIC value is considered best.

## Results

### Preliminary Analyses

Descriptive statistics for CDA dimensions (see Table 1) indicated that mean levels of perpetrated and suffered pressure-aggression and control-monitoring were quite low, although there was high variability. Repeated measures ANOVA and *post hoc* tests using the Bonferroni correction revealed that control-monitoring was significantly more common than pressure-aggression [F(1, 133) = 10.095, *p* = 0.002; η^2^ = 0.07] and that this difference was stronger for perpetrated than for suffered CDA [F(1, 133) = 9.130, *p* = 0.003; η^2^ = 0.06]. In addition, bivariate correlations among CDA dimensions (see Table 1) indicated that control-monitoring was more strongly correlated across partners (r = 0.52 and 0.69, for perpetrated and suffered CDA, respectively) than pressure-aggression (r = 0.33 and 0.29), suggesting a higher actual reciprocity in control-monitoring than in pressure-aggression. Similarly, perpetrated and suffered control-monitoring were more strongly correlated within partners (r = 0.69 and 0.66, for males and females, respectively) than perpetrated and suffered pressure-aggression (r = 0.42 and 0.44), indicating a higher perceived reciprocity in control-monitoring than in pressure-aggression. Also, correlational analyses revealed high inter-partner agreement on the occurrence of CDA (r’s ≥ 0.73), with the exception of pressure-aggression by males (r = 0.30).

**TABLE 1:** Descriptive and bivariate correlations among CDA dimensions.

	**1.**	**2.**	**3.**	**4.**	**5.**	**6.**	**7.**	**M**	**SD**	**Range**
1. M perpetrated pressure-aggression	–							0.10	0.19	0 – 1
2. F perpetrated pressure-aggression	0.33***	–						0.35	0.58	0 – 2.45
3. M perpetrated monitoring-control	0.39***	0.31***	–					1.66	1.34	0 – 5.22
4. F perpetrated monitoring-control	0.27**	0.61***	0.52***	–				1.36	1.48	0 – 5.78
5. M suffered pressure-aggression	0.42***	0.73***	0.43***	0.63***	–			0.27	0.48	0 – 2
6. F suffered pressure-aggression	0.30***	0.44***	0.26**	0.30***	0.29***	–		0.17	0.28	0 – 1.55
7. M suffered monitoring-control	0.39***	0.49***	0.69***	0.81***	0.65***	0.23**	–	1.17	1.43	0 – 5.11
8. F suffered monitoring-control	0.29***	0.46***	0.77***	0.66***	0.45***	0.40***	0.69***	1.53	1.39	0 – 5.11

M = males; F = females; CDA = Cyber Dating Abuse. *p < 0.05, **p < 0.01, ***p < 0.001.

When estimating within-dyad reciprocity and agreement through intraclass correlations (r_I_) (Kenny et al., 2006), we obtained similar patterns of results: reciprocity was higher for control-monitoring (r_I_ = 0.51, *p* = 0.000, and r_I_ = 0.67, *p* = 0.000, for perpetrated and suffered CDA respectively) than for pressure-aggression (r_I_ = 0.11, *p* = 0.190, and r_I_ = 0.24, *p* = 0.004) and inter-partner on the occurrence of CDA was high (r’s_I_ ≥ 0.71, *p* = 0.000), with the exception of pressure-aggression by males (r_I_ = 0.27, *p* = 0.001).

Multivariate regression analyses showed that all ACEs investigated were uniquely related with self-reported maladaptive schemas (abandonment and emotional deprivation) or with self-reported or partner-reported CDA, with the exception of males and females’ physical abuse which was therefore excluded from subsequent analyses (see Table 2). Also, regression analyses results were in line with the assumption that ACES could predict CDA not only within but also across partners. In fact, some ACEs reported by one partner – namely, emotional abuse, exposure to IPV by the opposite-sex parent, and (for males only) physical neglect – were significantly related to CDA reported by the other partner.

**TABLE 2:** Multivariate regression analyses predicting self-reported maladaptive schemas and self- and partner-reported CDA.

***Outcome Predictors***	**M abandonment**	**M emotional deprivation**	**M perpetrated pressure-aggression**	**F perpetrated pressure-aggression**	**M suffered pressure-aggression**	**F suffered pressure-aggression**	**M perpetrated monitoring-control**	**F perpetrated monitoring-control**	**M suffered monitoring-control**	**F suffered monitoring-control**
M Physical abuse	−0.05	13	0.17	0.05	−0.03	−0.09	0.00	−0.07	−0.07	0.17
M Emotional abuse	0.27	23*	−0.05	0.28	0.23	0.31*	0.30*	0.41**	0.50**	0.17
M Physical neglect	23	0.43***	0.30*	0.19	0.22	0.19	0.27*	0.02	0.18	0.35**
M Emotional neglect	−22	0.15	0.00	−0.14	−0.11	0.04	−0.41**	0.03	−0.28	−0.15
M Exposure to IPV by father	0.24*	−0.01	−0.04	0.20	0.04	0.15	−0.02	0.13	0.05	0.12
M Exposure to IPV by mother	−0.14	0.07	0.12	−0.20	0.04	−0.37**	0.32**	−0.06	0.11	−0.10

	**F abandonment**	**F emotional deprivation**	**M perpetrated pressure-aggression**	**F perpetrated pressure-aggression**	**M suffered pressure-aggression**	**F suffered pressure-aggression**	**M perpetrated monitoring-control**	**F perpetrated monitoring-control**	**M suffered monitoring-control**	**F suffered monitoring-control**

F Physical abuse	0.11	0.01	−0.20	0.13	0.16	0.02	−0.07	0.21	0.03	−0.05
F Emotional abuse	0.54**	0.51***	0.08	0.20	0.23	−0.42*	0.36*	0.27*	0.38**	0.42**
F Physical neglect	0.23	0.20*	0.22	0.15	0.01	0.10	0.21	0.05	0.14	0.31**
F Emotional neglect	−0.40*	0.18	0.16	−0.04	−0.01	0.20	−0.13	−0.12	−0.17	−0.23
F Exposure to IPV by father	−0.37**	−0.19	0.12	0.39**	0.42***	0.32*	0.10	0.39***	0.32**	0.11
F Exposure to IPV by mother	0.21	0.06	−0.11	−0.19	−0.09	0.04	0.05	−0.01	−0.05	0.07

M = males; F = females; CDA = Cyber Dating Abuse; IPV = Intimate Partner Violence. Standardized coefficients are reported. *p < 0.05, **p < 0.01, ***p < 0.001.

### APIM Models

#### ACEs → Emotional Deprivation → Perpetrated Pressure-Aggression

**FIGURE 2:**
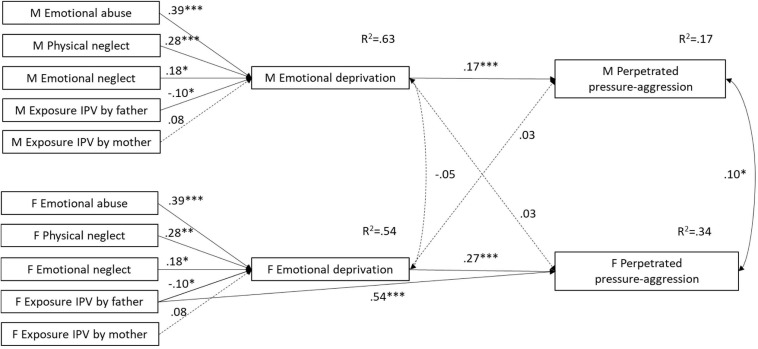
APIMeM with emotional deprivation as mediator and perpetrated pressure-aggression as outcome. M = males; F = females; IPV = Intimate Partner Violence. Standardized coefficients are reported. Correlations among ACEs are omitted from the figure for clarity. Model fit statistics: S-Bχ^2^(35) = 33.388, *p* = 0.497; R-CFI = 1.000; R-RMSEA = 0.000. ^∗^*p* < 0.05, ^∗∗^*p* < 0.01, ^∗∗∗^*p* < 0.001.

When individual and partner effects were constrained to be equal, the APIMeM positing emotional deprivation as mediator of the link between ACEs and perpetrated pressure-aggression yielded quite a good fit [S-Bχ^2^(37) = 68.8425, *p* = 0.001; R-CFI = 0.954; R-RMSEA = 0.080; R-AIC = −5.157]. However, the χ^2^ difference test indicated that the model fit could be significantly improved by allowing the paths from emotional deprivation to individual perpetrated pressure-aggression to be freely estimated across gender [S-B Δ*χ*^2^(1) = 9.739, *p* = 0.002]. Also, the LM test indicated that the model fit could be significantly improved by adding a direct path from females’ exposure to IPV acted by the father to individual perpetrated pressure-aggression [S-B Δ*χ*^2^(1) = 7.292, *p* = 0.007]. The final model had an excellent fit [S-Bχ^2^(35) = 33.388, *p* = 0.497; R-CFI = 1.000; R-RMSEA = 0.000] and explained a greater amount of variance in females’ (R^2^ = 0.34) than in males’ (R^2^ = 0.17) perpetrated pressure-aggression (see Figure 2). According to the model, emotional deprivation predicted pressure-aggression perpetrated by the respondent, but not pressure-aggression perpetrated by the partner; this individual effect was significantly stronger for females than for males. Also, for both males and females, emotional deprivation significantly mediated the association of emotional abuse and physical neglect with individual pressure-aggression, whereas emotional deprivation mediated the association between emotional neglect and individual pressure-aggression for males only (see Table 3). Finally, females’ exposure to IPV acted by the father strongly and directly predicted their perpetration of pressure-aggression.

**TABLE 3:** Indirect effects in the APIMeMs assuming emotional deprivation as mediator.

***Outcomes Predictors***	**M perpetrated pressure-aggression**	**F perpetrated pressure-aggression**	**M suffered pressure-aggression**	**F suffered pressure-aggression**	**M perpetrated monitoring-control**	**F perpetrated monitoring-control**	**M suffered monitoring-control**	**F suffered monitoring-control**
M Emotional abuse	0.07**	0.01	0.07	0.08*	0.08**	0.06*	0.08*	0.06*
M Physical neglect	0.05**	0.01	0.05	0.05*	0.06**	0.05*	0.06*	0.04*
M Emotional neglect	0.03*	0.01	0.03	0.04	0.04*	0.03	0.04*	0.03*
M Exposure to IPV by father	−0.02	0.00	−0.02	−0.02	−0.02	−0.02	−0.02	−0.01
M Exposure to IPV by mother	0.01	0.00	0.02	0.02	0.02	0.01	0.02	0.01
F Emotional abuse	0.01	0.11*	0.08*	−0.02	0.06*	0.08**	0.06*	0.08*
F Physical neglect	0.01	0.08*	0.05*	−0.01	0.05*	0.06**	0.04*	0.06*
F Emotional neglect	0.01	0.05	0.04	−0.01	0.03	0.04*	0.03*	0.04*
F Exposure to IPV by father	0.00	−0.03	−0.02	0.00	−0.02	−0.02	−0.01	−0.02
F Exposure to IPV by mother	0.00	0.02	0.02	0.00	0.01	0.02	0.01	0.02

M = males; F = females; IPV = Intimate Partner Violence. Standardized coefficients are reported. *p < 0.05, **p < 0.01, ***p < 0.001.

#### ACESs → Emotional Deprivation → Suffered Pressure-Aggression

**FIGURE 3:**
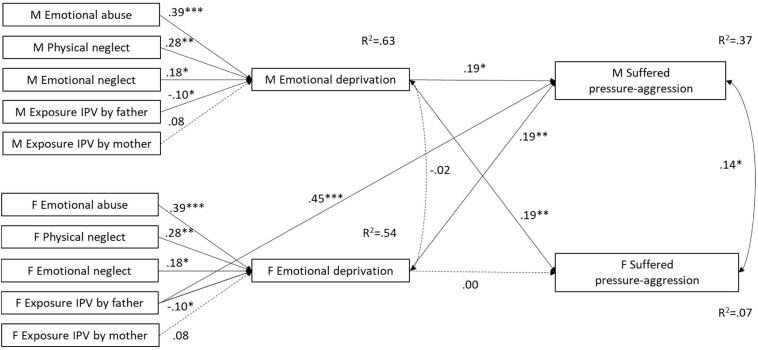
Abime with emotional deprivation as mediator and suffered pressure-aggression as outcome. M = males; F = females; IPV = Intimate Partner Violence. Standardized coefficients are reported. Correlations among ACEs are omitted from the figure for clarity. Model fit statistics: S-Bχ^2^(35) = 49.986, *p* = 0.048; R-CFI = 0.979; R-RMSEA = 0.057. ^∗^*p* < 0.05, ^∗∗^*p* < 0.01, ^∗∗∗^*p* < 0.001.

When individual and partner effects were constrained to be equal, the APIMeM in which ACEs were assumed to predict suffered pressure-aggression through emotional deprivation did not yield an adequate fit [S-Bχ^2^(37) = 90.441, *p* = 0.000; R-CFI = 0.924; R-RMSEA = 0.105; R-AIC = 16.941]. The model fit significantly improved when the path from emotional deprivation to individually suffered pressure-aggression was freely estimated across gender [S-B Δ*χ*^2^(1) = 18.588, *p* = 0.000], and when a direct path from females’ exposure to IPV acted by the father to partner suffered pressure-aggression was added to the model [S-B Δ*χ*^2^(1) = 14.522, *p* = 0.000]. The final model had a good fit [S-Bχ^2^(35) = 49.986, *p* = 0.048; R-CFI = 0.979; R-RMSEA = 0.057] and explained a greater amount of variance in males (R^2^ = 0.37) than in females (R^2^ = 0.07) suffering pressure-aggression (see Figure 3). According to the model, emotional deprivation was related to pressure-aggression suffered by the partner for both males and females as well as to pressure-aggression suffered by respondents for males only. Also, for both males and females, emotional deprivation significantly mediated the association of emotional abuse and physical neglect with pressure-aggression suffered by the partner (see Table 3). Finally, females’ exposure to IPV acted by the father strongly and directly predicted pressure-aggression suffered by their partner.

#### ACEs → Emotional Deprivation → Perpetrated Control-Monitoring

**FIGURE 4:**
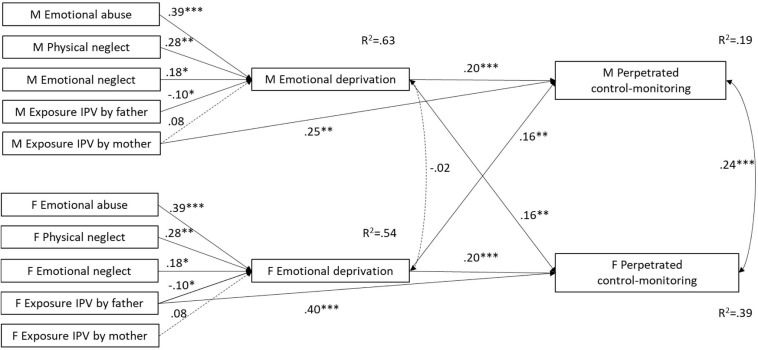
APIMeM with emotional deprivation as mediator and perpetrated control-monitoring as outcome. M = males; F = females; IPV = Intimate Partner Violence. Standardized coefficients are reported. Correlations among ACEs are omitted from the figure for clarity. Model fit statistics: S-Bχ^2^(35) = 61.838, *p* = 0.003; R-CFI = 0.962; R-RMSEA = 0.076. ^∗^*p* < 0.05, ^∗∗^*p* < 0.01, ^∗∗∗^*p* < 0.001.

When individual and partner effects were constrained to be equal, the APIMeM positing that ACEs predict perpetrated control-monitoring through emotional deprivation did not yield an adequate fit [S-Bχ^2^(37) = 95.751, *p* = 0.000; R-CFI = 0.917; R-RMSEA = 0.109; R-AIC = 21.751]. Equality constraints on individual and partner parameters were correctly imposed, indicating that dyad members did not differ in this regard. However, the LM test suggested that the model fit could be significantly improved by adding a direct path from females’ exposure to IPV by the father to individual perpetrated control-monitoring [S-B Δ*χ*^2^(1) = 14.181, *p* = 0.000] as well a direct path from males’ exposure to IPV by the mother to individual perpetrated control-monitoring [S-B Δ*χ*^2^(1) = 10.172, *p* = 0.001]. The final model had an adequate fit [S-Bχ^2^(35) = 61.838, *p* = 0.003; R-CFI = 0.962; R-RMSEA = 0.076] and explained a greater amount of variance in females’ (R^2^ = 0.39) than in males’ (R^2^ = 0.19) perpetrated control-monitoring (see Figure 4). According to the model, emotional deprivation predicted perpetrated control-monitoring both within and across partners. Emotional abuse and physical neglect indirectly predicted control-monitoring perpetrated by both the individual and his/her partner, whereas emotional neglect indirectly predicted control-monitoring perpetrated by the individual only. Exposure to IPV enacted by opposite-sex parents directly predicted control-monitoring perpetrated by the respondent for both males and females.

#### ACEs → Emotional Deprivation → Suffered Control-Monitoring

**FIGURE 5:**
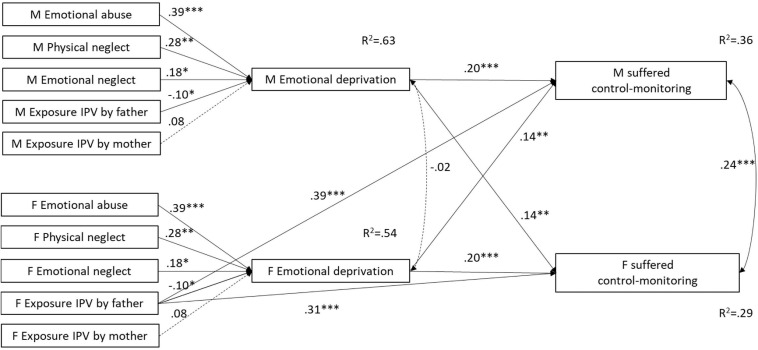
APIMeM with emotional deprivation as mediator and suffered control-monitoring as outcome. M = males; F = females; IPV = Intimate Partner Violence. Standardized coefficients are reported. Correlations among ACEs are omitted from the figure for clarity. Model fit statistics: S-Bχ^2^(35) = 56.182, *p* = 0.013; S-B CFI = 0.970; RMSEA = 0.067. ^∗^*p* < 0.05, ^∗∗^*p* < 0.01, ^∗∗∗^*p* < 0.001.

When individual and partner effects were constrained to be equal, the APIMeM in which ACEs predicted suffered control-monitoring through emotional deprivation did not result in an adequate fit [S-χ^2^(37) = 76.419, *p* = 0.000; S-B CFI = 0.944; RMSEA = 0.090; R-AIC = 2.419]. Equality constraints on individual and partner parameters were correctly imposed, indicating that dyad members did not differ in this regard. However, the LM test suggested that the model fit could be significantly improved by adding direct paths from females’ exposure to IPV by the father to both individual and partner suffered control-monitoring [S-B Δ*χ*^2^(1) = 19.822, *p* = 0.000 for the individual path; S-B Δ*χ*^2^(1) = 11.666, *p* = 0.001 for the partner path]. The final model had an adequate fit [S-Bχ^2^(35) = 56.182, *p* = 0.013; S-B CFI = 0.970; RMSEA = 0.067] and explained a similar amount of variance in males (R^2^ = 0.36) and females (R^2^ = 0.29) suffering control-monitoring (see Figure 5). According to the model, emotional deprivation predicted suffered control-monitoring both within and across partners. The indirect effects of emotional abuse, emotional neglect, and physical neglect on control-monitoring suffered by the respondent and by his/her partner were all significant. Females’ exposure to IPV by the father directly predicted control monitoring suffered by both themselves and their partner.

#### ACEs → Abandonment → Perpetrated Pressure-Aggression

The APIMeM testing abandonment as a mediator of the association between ACEs and perpetrated pressure-aggression yielded a very poor fit [S-Bχ^2^(37) = 118.633, *p* = 0.000; R-CFI = 0.869; R-RMSEA = 0.129; R-AIC = 44.633], due to the lack of significant relationships between abandonment and pressure-aggression perpetrated by the respondent or by his/her partner for both males and females.

#### ACEs → Abandonment → Suffered Pressure-Aggression

The APIMeM in which abandonment mediates the association between ACEs and suffered pressure-aggression also yielded a very poor fit [S-Bχ^2^(37) = 127.976, *p* = 0.000; R-CFI = 0.856; R-RMSEA = 0.136; R-AIC = 53.976], because abandonment was unrelated to individual suffered pressure-aggression for females and to partner suffered pressure-aggression for both males and females. The model did not achieve a satisfactory fit even when, consistent with the assumption of a partial mediation model, direct paths from ACEs to individual and partner suffered pressure-aggression were added.

#### ACEs → Abandonment → Perpetrated Control-Monitoring

The APIMeM positing that ACEs predict perpetrated control-monitoring through abandonment yielded a very poor fit [S-Bχ^2^(37) = 152.567, *p* = 0.000; R-CFI = 0.820; RMSEA = 0.153; R-AIC = 78.566], probably due to the fact ACEs were related to perpetrated control-monitoring directly rather than indirectly. However, the model fit remained unsatisfactory even when, consistent with a partial mediation model, direct paths from ACEs to individual and partner perpetrated control-monitoring were added.

#### ACES → Abandonment → Suffered Control-Monitoring

The APIMeM in which ACEs predict suffered control-monitoring through abandonment also resulted in a poor fit [S-Bχ^2^(37) = 132.515, *p* = 0.000; R-CFI = 0.851; R-RMSEA = 0.139; R-AIC = 58.515]. The fit was not significantly improved by the introduction of direct paths from ACEs to control-monitoring suffered by the respondent or by his/her partner for both males and females.

#### Alternative Models

Finally, since the cross-sectional nature of our data cannot rule out the possibility of reverse effects between maladaptive schemas and CDA dimensions, alternative APIMeMs in which outcomes and mediators were reversed were also tested. The alternative APIMeMs positing CDA dimensions as mediators of the association between ACEs and emotional deprivation yielded much poorer fits than our hypothesized APIMeMs, due to the fact ACEs were related to emotional deprivation directly rather than indirectly (CFIs ≤ 0.790; R-RMSEA ≥ 0.187; Δ R-AICs ≥ 113.893; see Supplementary Materials for details).

On the contrary, alternative APIMeMs testing CDA dimensions as mediators of the association between ACEs and abandonment yielded equal or better fits than our hypothesized APIMeMs (Δ R-AICs ≤ 0.186). However, the fits for these alternative models, like those for our hypothesized models, were far from acceptable (CFIs ≤ 0.886; R-RMSEA ≥ 0.121; see Supplementary Materials). The poor fits for APIMeMs assuming pressure-aggression as a mediator was due to the lack of significant relationships between pressure-aggression and abandonment, which had been already observed in our hypothesized models. Surprisingly, the poor fits for APIMeMs testing control-monitoring as a mediator seemed mainly due to a lack of direct paths from ACEs to CDA, yet the introduction of these paths did not improve model fits enough to become acceptable.

## Discussion

This study investigated CDA among young adults using the romantic couple as the unit of analysis and taking into account the interdependence of its members. Specifically, informed by Schema Therapy and IPV research, the study posited that ACEs predicted perpetrated and suffered CDA both within and across partners through the mediation of emotional deprivation and abandonment schemas.

### CDA Frequency and Reciprocity

Our preliminary analyses showed that, even though not particularly frequent, CDA occurs in most young adult romantic couples, at least in the form of persistent control and monitoring behaviors. Indeed, both partners agree that control-monitoring was a more frequent form of CDA than pressure aggression, although this prevalence was more strongly perceived by abuse perpetrators than by their victims. Also, cyber dating control-monitoring was characterized by more reciprocity than cyber dating pressure-aggression both within and across partners. These data are consistent with prior CDA findings and can be explained by the fact that adolescents and young adults judge controlling behaviors as less serious and abusive than aggressive or sexting behaviors (e.g., Zweig et al., 2013, 2014; Borrajo et al., 2015a,b,c; Reed et al., 2017), which could lead them to enacting and reporting these behaviors more easily. Also, according to some sociologists (Acquisti and Gross, 2006; Hallam and Zanella, 2017; Koohikamali et al., 2017), today’s society, full of stimuli and invitations to exhibit the self, creates a sort of short circuit between the need or pleasure of having an attractive partner and the fear of being betrayed by him or her. To cope with this fear, adolescents and young adults tend therefore to control their partner more assiduously. Finally, an explanation for the higher levels of acted, than suffered, control-monitoring could be found in the fact that control is a form of violence that the victim may not be aware of.

### Within and Across Partners Associations of ACEs With Cyber Dating Pressure-Aggression Through Emotional Deprivation

Our APIMeM analyses showed that having experienced family emotional abuse and physical neglect during childhood and adolescence indirectly increased in both males and females the likelihood of perpetrating cyber dating pressure and aggression by increasing the internalization of the emotional deprivation schema. These findings were supported by both self-reported and partner-reported data and are in line with research showing that similar experiences lead people to be more aggressive in romantic relationships (Garrido and Taussig, 2013; Karakurt et al., 2013; Iverson et al., 2014; Eriksson and Mazerolle, 2015; Atmaca and Gencoz, 2016; Machisa et al., 2016; Smith-Darden et al., 2016; Madruga et al., 2017; Ramos et al., 2017; Kimber et al., 2018). Furthermore, an indirect effect on perpetrated cyber dating pressure and aggression was found for physical neglect experienced by males. No direct or indirect effects were found for physical abuse and witnessed intimate partner violence enacted by the same-sex parent of the respondent. It is possible that physical abuse shows greater impacts on traditional physical violence rather than on digital aggression and that seeing the same sex-parent perpetrating intimate partner violence does not lead the person to identify himself/herself with an emotionally deprived and dissatisfied figure, thereby not causing the internalization of this schema.

The APIMeM analyses also showed that, independently of their childhood experiences, the more couple members had internalized the belief that people will never be able to fully satisfy their needs for care, affection, and relational stability, the more they perpetrated online psychological or sexual pressure and aggression against their partner and the more their partner acknowledged having suffered these forms of abuse. Significant gender differences emerged: the internalization of the emotional deprivation schema was more strongly related to perpetrated pressure-aggression for females and to suffered pressure-aggression for males. Despite these differences in strength, self-reported and partner-reported data were consistent in indicating that young adults were more likely to enact cyber dating pressure and aggression if they felt emotionally deprived by others. The findings can be explained using the theory of early maladaptive patterns (Young et al., 2007), according to which the coping style based on overcompensation, which for subjects with emotional deprivation is associated with the tendency to be excessively demanding on the partner to satisfy their emotional needs, explain the propensity to commit aggressive acts (Young and Flanagan, 1998). The findings are also consistent with the idea that emotional deprivation makes people more sensitive to identifying behaviors that confirm their belief that they are not satisfied with the relationship. However, the fact that this greater sensitivity was found mostly in males could be ascribed to the fact that psychological or sexual pressure and aggression are more socially accepted when performed by men. Therefore, being male and feeling a victim of this form of violence could be experienced as even more serious and harmful (West and Fenstermaker, 1995; Anderson, 1997).

Finally, a strong and direct association was found between females’ exposure to violence by the father against the mother and females’ tendency to pressure and be aggressive online toward their male partner, an association which was confirmed when considering pressure-aggression reported by the male partner. Thus, it seems that having suffered this adverse early experience led females to replicate online the abusing behaviors they observed offline in their father, perhaps as a way to protect themselves from the possibility of reliving what their mother suffered.

### Within and Across Partners Associations of ACEs With Cyber Dating Control-Monitoring Through Emotional Deprivation

APIMeM results showed that the likelihood of perpetrating and suffering cyber dating control and monitoring was indirectly predicted in both males and females having experienced family emotional abuse, physical neglect, and emotional neglect during childhood and adolescence, through the internalization of the emotional deprivation schema. The only case in which emotional neglect was not predictive of control-monitoring was when partner effects for perpetrated control-monitoring were considered. Similar to what emerged for pressure-aggression, no direct or indirect effects were found for physical abuse and witnessed violence by the same-sex parent of the respondent. All these findings were supported by both self-reported and partner-reported data and are consistent with the Schema Therapy and IPV literature previously reviewed.

Our APIMeM also showed that, independently of their adverse child experiences, the more couple members had internalized the emotional deprivation schema, the more they perpetrated and suffered cyber dating control and monitoring. Moreover, the more one partner perpetrated control against the other, the more the latter acknowledged having suffered this form of violence and reacted to this behavior in kind. No gender differences emerged, probably both because control behaviors are perceived as more acceptable for females and because, compared to aggressive behaviors, control behaviors are characterized by greater interdependence between partners (Zweig et al., 2013). Therefore, people believing that they cannot be fully satisfied in their emotional and relational need by anyone are not only prone to verifying the trust granted to the other, but also probably enact behaviors that induce the partner to perform the same type of controlling behaviors. People affected by Borderline Personality Disorder, for example, tend to manifest behaviors that make the partner jealous and lead the partner to control them, and this personality disorder is etiologically attributable to experiences such as those underlying the internalization of the emotional deprivation scheme (DSM-V; Selvini, 2017).

Finally, a strong and direct association was found between males and females’ exposure to intimate partner violence perpetrated by the opposite-sex parent and their tendency to control and monitor the partner online. These results parallel the previous ones related to pressure-aggression and could be interpreted as a strategy not to assume in the current romantic relationship the victim role the same-sex parent had in their family of origin.

### Within and Across Partners Associations of ACEs With Cyber Dating Pressure-Aggression and Control-Monitoring Through Abandonment

The abandonment schema was unrelated to cyber dating pressure-aggression, nor did it mediate the relationship between any of the adverse childhood experiences investigated and CDA. This finding was quite unexpected, since people who believe they are always dealing with unpredictable and lying partners, ready to invest in other relationships, could be assumed to control them (Young et al., 2007). However, the theory of maladaptive schemas offers a possible interpretation of this result: the adoption of a coping style based on surrender could induce individuals who have internalized this schema not to invest in deep relationships in order to counter the onset of a morbid attachment toward the partner (Young et al., 2007). Also, it is possible that the absence of significant results relating to this scheme was partly due to the small sample size.

### Limitations and Conclusions

Several limitations of the study should be considered when interpreting these results.

First, because it used a cross-sectional design, inferences regarding direction of effects cannot be drawn with confidence. Even though alternative models tested provided additional evidence supporting the proposed role of emotional deprivation as mediator between ACEs and CDA, even this evidence is too inconclusive to uncover causal relations because of its cross-sectional nature.

Second, the size of the sample and the choice to recruit non-cohabiting couples through posts on Facebook pages dedicated to university groups, which led mostly self-selected highly educated students to participate in the study, threaten the generalizability of our results to other types of subjects and couples. Since multi-problem families with high levels of ACEs and violence often have low levels of education (Asen et al., 2001; Asen and Scholz, 2010), we cannot rule out the possibility that our results might differ in less educated samples. However, given the fact that most international research on cyber dating abuse is based on samples consisting solely or predominantly of psychology students, this study has the merit of investigating students enrolled in a wide range of faculties, both humanistic and scientific. In addition, workers and unemployed people, even if numerically lower than students (8.7% of females and 29.7% of males), were not entirely absent from the sample.

Third, only two of the five schemas belonging to the disconnection and rejection domain (that is emotional deprivation and abandonment schemas) were analyzed, because the Italian validation of the Young Schema Questionnaire Short Form (Celsi, 2019, unpublished) yielded only these two factors.

Lastly, this study did not investigate whether partnership variables, such as the levels of satisfaction, investment, commitment, trust, and quality of alternatives, would influence or possibly buffer the relationships found.

Future studies could try to overcome some of these limits by investigating CDA predictors through longitudinal designs, expanding the sample to adolescent couples and to adult romantic couples (both cohabiting and non-cohabiting and highly and poorly educated), using the Young Schema Questionnaire Long Form, which has been validated for use in Italian samples (Saggino et al., 2014), and taking into account the possible predictive or moderating role of partnership variables.

Notwithstanding the limitations noted above, this study made significant contributions to the emerging literature on CDA. First, it has the merit of documenting whether some predictors of IPV, specifically ACEs and early maladaptive schemas, also predict CDA. In this regard, the study provides initial evidence that emotional abuse, physical neglect, emotional neglect, and the schema of emotional deprivation play a similar predictive role in relation to CDA. This supports the idea of close similarities between the two forms of couple violence. At the same time, the lack of a significant link between CDA and physical abuse, which has been found to be a distal predictor of IPV (McKinney et al., 2009; Iverson et al., 2014; Widom et al., 2014; Machisa et al., 2016; Voith et al., 2017), suggests that CDA may also function differently from IPV in some ways. A similar conclusion is suggested by the lack of a significant association between CDA and the schema of abandonment when controlling for adverse early childhood experiences. Despite the paucity of research on this specific schema, several studies have provided evidence to show that schemas belonging to the Disconnection and rejection domain predict IPV (e.g., Gay et al., 2013; Falahatdoost et al., 2014; Taşkale and Soygüt, 2017). Therefore, it seems necessary to investigate further the unique role that the abandonment schema has in predicting IPV and CDA. Additional study of the relationship between CDA and witnessed intimate partner violence perpetrated by the opposite-sex parent also deserves attention. In the present study, witnessing violence by the opposite-sex parent was particularly predictive of perpetrated CDA for females.

Second, the study has the merit of investigating CDA from a dyadic perspective, evaluating the effects of both partners’ early adverse experiences and maladaptive schemas, and doing so when partners were considered in their dual roles as perpetrators and victims. This perspective yielded new, interesting results, such as those showing that each partner’s individual early experiences and schemas are likely to predict not only their own tendency to overcontrol their romantic partner but also their partner’s tendency to overcontrol them. The use of both self-report and partner report was also important, as it revealed a convergence of results between the perspective of the victim and that of the offender.

Finally, gaining a better understanding of the predictive role that specific ACEs and early maladaptive schemas exert on the likelihood of perpetrating and suffering cyber dating abuse appears useful for both preventive and clinical programs. More specifically, regarding prevention, recent years have seen the spread of bystander programs aimed at combating dating violence (Storer et al., 2016). These programs aim to help young people to develop the skills to recognize violent acts and intervene when they witness behavior that can lead to violence. Designed to prevent sexual violence, these programs were then extended to other forms of violence (e.g., psychological violence and control) and proved to be effective because they counteract the tacit reinforcement that violence receives from the fact that peers ignore, and therefore substantially endorse, violent behavior (Katz et al., 2011). For example, being aware that a friend checks his/her romantic partner’s geolocation without the partner’s knowledge, and not expressing dissent toward this behavior, creates implicit reinforcement. On the contrary, expressing disappointment and trying to make the friend reflect on the negative aspects of the act can increase awareness and generate doubts about the acceptability of the behavior. However, these types of intervention, undoubtedly important on a social level, may be insufficient to trigger a profound change in those most prone to commit violence. Therefore, we believe that second-level interventions, addressed precisely to the subjects most at risk, can be best constructed only by having a more specific and in-depth knowledge of the background and personality characteristics of these same subjects. Similarly, from a clinical point of view, knowing which adverse experiences and which early maladaptive schemas are most connected to violence could facilitate the patient’s cognitive restructuring work. According to the Schema Therapy framework (Young et al., 2007), this work involves cognitive strategies aimed at helping the patient to identify situations that disconfirm the internalized maladaptive schemas, as well as experiential techniques based on imagination or role-playing exercises which lead patients to focus on and counter the anger and sadness connected to childhood adverse experiences. These cognitive and experiential strategies could be better designed and implemented in clinical interventions for CDA couples, if the specific early adverse experiences and maladaptive schemas which foster CDA are known. Moreover, deepening knowledge of similarities and differences between IPV and CDA predictors could be particularly useful for understanding whether prevention activities carried out to reduce offline couple violence are also suitable to counter online couple violence or whether specific programs are needed for IPV and CDA.

## Data Availability Statement

The raw data supporting the conclusions of this article will be made available by the authors, upon justified request.

## Ethics Statement

Ethical review and approval was not required for the study on human participants in accordance with the local legislation and institutional requirements. The participants provided their written informed consent to participate in this study.

## Author Contributions

LC and FGP designed the study, LC collected the data, and LC and FGP analyzed them. All co-authors participated in the discussion of the results, drafted the manuscript and approved it for publication.

## Conflict of Interest

The authors declare that the research was conducted in the absence of any commercial or financial relationships that could be construed as a potential conflict of interest.

## Footnotes

1 Three items were not invariant across gender for pressure-aggression (“My partner said things to my friends through SMS/emails/social networks to turn them against me,” “My partner pressured me to send him/her photos of me naked or in sexually explicit poses,” “My partner pressured me to do sexting”) and three were not invariant for control-monitoring (“My partner made me delete from my friends list the accounts of people he/she considered inappropriate,” “My partner wanted to know and monitored who my friends are on social networks,” “My partner pressured me to respond quickly to his/her calls, SMS or other messages”). Specifically, the last two control-monitoring items were not invariant when assessing suffered CDA, whereas the other four items were not invariant when assessing perpetrated CDA.
