# Supplementary material for: Adverse Childhood Experiences and Early Maladaptive Schemas as Predictors of Cyber Dating Abuse: An Actor-Partner Interdependence Mediation Model Approach
Source: Front Psychol. 2021 Mar 18;12:623646. doi: 10.3389/fpsyg.2021.623646 (PMC8012817; doi:10.3389/fpsyg.2021.623646)
Supplement: Supplementary file 1 [file Table_1.DOCX]

**Supplementary Materials**

**Supplementary Table 1.** Correlations among ACEs resulting from APIMeMs

|  | 1 | 2 | 3 | 4 | 5 | 6 | 7 | 8 | 9 |
| --- | --- | --- | --- | --- | --- | --- | --- | --- | --- |
| 1. M Emotional abuse | - |  |  |  |  |  |  |  |  |
| 2. M Physical neglect | .64*** | - |  |  |  |  |  |  |  |
| 3. M Emotional neglect | .77*** | .76*** | - |  |  |  |  |  |  |
| 4. Exposure to IPV by father | .31** | .18 | .32** | - |  |  |  |  |  |
| 5. M Exposure to IPV by mother | .54*** | .31** | .50*** | .58*** | - |  |  |  |  |
| 6 Emotional abuse | .37** | .33** | .31** | .16 | .18 | - |  |  |  |
| 7. F Physical neglect | .38*** | .37** | .37** | .33** | .24** | .76*** | - |  |  |
| 8. F Emotional neglect | .30** | .31** | .31** | .20* | .12 | .71*** | .70*** | - |  |
| 9. Exposure to IPV by father | .41*** | .50*** | .41*** | .20* | .21* | .56*** | .58*** | .53*** | - |
| 10. Exposure to IPV by mother | .26** | .32** | .32** | .05 | .21* | .66*** | .40*** | .62*** | .56*** |

*Note*. M=males; F=females; IPV=Intimate Partner Violence. Standardized coefficients are reported.

*p<.05, **p<.01, ***p<.001

**Supplementary Table 2.** Model fit statistics for alternative models

| Alternative model | S-Bχ2 | df | p | R-CFI | R-RMSEA | R-AIC | Δ R-AIC  with respect to the hypothesized model |
| --- | --- | --- | --- | --- | --- | --- | --- |
| *ACEs → Emotional deprivation → Perpetrated pressure-aggression* | 268.509 | 37 | .000 | .790 | .217 | 128.115 | 133.272 |
| *ACEs → Emotional deprivation → Suffered pressure-aggression* | 220.966 | 37 | .000 | .739 | .193 | 146.966 | 130.025 |
| *ACEs → Emotional deprivation → Perpetrated control-monitoring* | 209.644 | 37 | .000 | .757 | .187 | 135.644 | 113.893 |
| *ACEs → Emotional deprivation → Suffered control-monitoring* | 216.002 | 37 | .000 | .745 | .191 | 142.002 | 139.583 |
| *ACEs → Abandonment → Perpetrated pressure-aggression* | 109.067 | 37 | .000 | .884 | .121 | 35.067 | -9.915 |
| *ACEs → Abandonment → Suffered pressure-aggression* | 128.162 | 37 | .000 | .856 | .136 | 54.162 | 0.186 |
| *ACEs → Abandonment → Perpetrated control-monitoring* | 110.139 | 37 | .000 | .886 | .122 | 36.139 | -42.427 |
| *ACEs → Abandonment → Suffered control-monitoring* | 112.283 | 37 | .000 | .883 | .124 | 38.283 | -.20.232 |

*Note*. ACEs= Aversive Childhood Experiences.

*p<.05, **p<.01, ***p<.001
